# Injectable Gel Form of a Decellularized Bladder Induces Adipose-Derived Stem Cell Differentiation into Smooth Muscle Cells In Vitro

**DOI:** 10.3390/ijms21228608

**Published:** 2020-11-15

**Authors:** Victoria Moreno-Manzano, Daria Zaytseva-Zotova, Eric López-Mocholí, Álvaro Briz-Redón, Berit Løkensgard Strand, Ángel Serrano-Aroca

**Affiliations:** 1 Neuronal and Tissue Regeneration Lab, Centro de Investigación Príncipe Felipe, c/Eduardo Primo Yúfera, 3, 46012 Valencia, Spain; elopezm@cipf.es; 2 NOBIPOL, Department of Biotechnology and Food Science, NTNU Norwegian University of Science and Technology, Sem Sælands vei 6-8, N-7491 Trondheim, Norway; daria.zaytseva-zotova@ntnu.no (D.Z.-Z.); berit.l.strand@ntnu.no (B.L.S.); 3 Statistics Office, City Council of Valencia, Plaza Ayuntamiento 1, 46002 Valencia, Spain; Alvaro.Briz@uv.es; 4 Biomaterials and Bioengineering Lab, Centro de Investigación Traslacional San Alberto Magno, Universidad Católica de Valencia San Vicente Mártir, c/Guillem de Castro 94, 46001 Valencia, Spain

**Keywords:** gel, extracellular matrix, viscoelasticity, rheology, scaffold, pepsin, adipose-derived stem cells, smooth muscle cells, cell differentiation, Matrigel

## Abstract

Biologic scaffolds composed of extracellular matrix components have been proposed to repair and reconstruct a variety of tissues in clinical and pre-clinical studies. Injectable gels can fill and conform any three-dimensional shape and can be delivered to sites of interest by minimally invasive techniques. In this study, a biological gel was produced from a decellularized porcine urinary bladder by enzymatic digestion with pepsin. The enzymatic digestion was confirmed by visual inspection after dissolution in phosphate-buffered saline solution and Fourier-transform infrared spectroscopy. The rheological and biological properties of the gel were characterized and compared to those of the Matrigel^TM^ chosen as a reference material. The storage modulus G’ reached 19.4 ± 3.7 Pa for the 30 mg/mL digested decellularized bladder gels after ca. 3 h at 37 °C. The results show that the gel formed of the porcine urinary bladder favored the spontaneous differentiation of human and rabbit adipose-derived stem cells in vitro into smooth muscle cells to the detriment of cell proliferation. The results support the potential of the developed injectable gel for tissue engineering applications to reconstruct for instance the detrusor muscle part of the human urinary bladder.

## 1. Introduction

Stem-cell therapies have been shown to be a promising approach to enhance the regeneration of a tissue-engineered bladder [1,2]. Adipose tissue-derived mesenchymal stem cells (ADSC) among other different stem cells are easily and abundantly obtained from diverse adult bodies with a low immunogenicity for potential autologous applications and they have already shown positive effects on the urethral tract functions in experimental animals [3], a model of neobladder reconstruction by recellularized acellular matrix bladder [4] and on porous synthetic scaffolds [5,6]. ADSC possess the traits of multilineage differentiation potential and ease of isolation, representing a promising resource for tissue engineering and regenerative medicine in urology to promote regeneration of smooth muscle, neural cells or even blood vessels when included in appropriated scaffolding material [5]. Furthermore, ADSC have shown to be a promising source to induce urothelial cells to form the urothelium when ADSC are cultured in an urothelial-conditioned medium [7]. Thus, ADSC have recently shown potential in the treatment of urinary incontinence [8]. Moreover, the safety and feasibility of ADSC therapy in the urothelial tracts in humans has already been demonstrated [9]. However, the underlying mechanisms of action remain controversial. Increasing evidence defends the paracrine effect rather than cell differentiation as the main factor for regenerating the functional tissues [10]. We recently demonstrated that the decellularized bladder matrix repopulated with ADSC could be a very promising translational strategy for complete cystectomies [11]. The urinary bladder is a dynamic organ that must be capable of allowing urine storage and at the same time be contractile to allow coordinated emptying. Its physiologic properties derived from the anatomic properties of the bladder smooth muscle and its mechanical behavior are dependent on the constituent properties of the extracellular matrix (ECM) and SMCs [12]. SMCs in the urinary bladder wall passively affect the mechanical behavior during refilling [13] and are responsible for contraction of the bladder during micturition as a result of active force generation.

ADSC are considered a possible source of SMCs in the regeneration of muscular layer of urinary bladder wall due to their impressive differentiation potential. Thus, they have been cultured in the presence of inductive media containing differentiation-promoting agents and growth factors [14,15,16,17,18] and subsequently the differentiated cells can be seeded onto a decellularized scaffold [19]. Thus, the specific differentiation of human ADSC into SMCs was always stimulated in inductive media. Beside ECM proteins, several factors contribute to smooth muscle cell development and differentiation, e.g., transforming growth factor (TGF)-β treatment results in the upregulation of contractile proteins in both stem/progenitor cells and SMCs [20,21,22]. However, as far as we know, the direct differentiation of human ADSC into SMCs induced by the scaffold material without induction media has never been explored. 

The ECM is made up of two main classes of macromolecules: (1) fibrous proteins, including collagen, elastin, fibronectin and laminin; and (2) glycosaminoglycans covalently bound to proteins forming proteoglycans [23]. ECM mimetic scaffolds are being widely used in biomedical applications and major challenges are to engineer ECM-like gels that degrade in response to appropriate ambient stimuli in the body and to make them biocompatible to avoid immune system reactions [24,25,26]. Many gels have been derived from ECM components such as collagen, hyaluronic acid and elastin or complex mixtures of ECM proteins that resemble the complex extracellular environment found in many tissues such as Matrigel [27,28], which is the trade name for a gelatinous protein mixture secreted by Engelbreth–Holm–Swarm mouse sarcoma cells [29,30]. However, although these gels are very useful for many cell culture applications, ECM scaffolds produced from decellularized tissues retain the full biochemical complexity of native tissue, and, unlike Matrigel, they are not composed of a protein source that is the product of a tumorigenic cell line. Thus, ECM bioscaffolds prepared from decellularized tissues have already been used to facilitate constructive and functional tissue remodeling in a variety of clinical applications generated from cadaveric tissues [31]. In vitro [32] and in vivo [33] applications have been developed as culture substrates comparable to collagen or Matrigel and as injectable materials for irregular tissue reconstruction. ECM decellularized scaffolds retain the nanostructure and bioinductive properties of the native matrix, and they have been shown to promote the in vivo creation of site-specific, functional tissue because of the preservation of the structural and functional proteins of the naïve tissue such as glycosaminoglycans, proteoglycans and growth factors [34]. In this regard, gels derived from the porcine urinary bladder matrix (UBM) produced by pepsin-solubilization have been shown to retain the inherent bioactivity of the matrix with the ability to support adhesion and growth of SMCs when cultured under static in vitro conditions promoting constructive remodeling in tissue engineering [35]. Pepsin is an endopeptidase, which is one of three principal proteolytic enzymes in the digestive system, produced in the mucosal lining of the stomach, and its function is to break down proteins to their components (peptides and amino acids) which can be readily absorbed by the intestinal surface proteins during the process of digestion [36]. Pepsin has been previously used also to extract collagen from skin and swim bladder of grass carp [37] or swim bladders of yellowfin tuna [38]. This type of gels can be used as injectable ECM gels with appropriate viscosities for delivery in three-dimensional spaces via minimally invasive surgical methods [27].

Since collagen-like scaffolds have shown the ability to induce in vitro and in vivo stem cell differentiation of bone-marrow cells, mesenchymal stem cells and placenta-derived multipotent cells into osteoblasts, chondrocytes and SMCs [39,40], respectively, mediated by collagen–α2β1 integrin interaction [41], the hypothesis of this work consisted of preparing a biological gel form of the porcine urinary bladder as scaffold with adequate biocompatible and rheological properties able to induce human and rabbit ADSC differentiation into SMCs without using induction media. In addition, this induction capacity of differentiation was compared with the commercial Matrigel^TM^, which can be used as reference gel because it contains growth factors, such as TGF-β and EGF, that promote proliferation and/or differentiation, and it is also mainly composed of ECM structural proteins such as laminin, nidogen, collagen and heparan sulfate proteoglycans [29]. The use of decellularized based gels for cell therapy could be a very promising strategy to efficiently repair damaged irregular tissues in an injectable form avoiding additional use of growth factors, reducing immunogenic reactions, risk of cancer and alterations in cellular homeostasis [42,43].

## 2. Results and Discussion

### 2.1. Material Characterization

**Figure 1:**
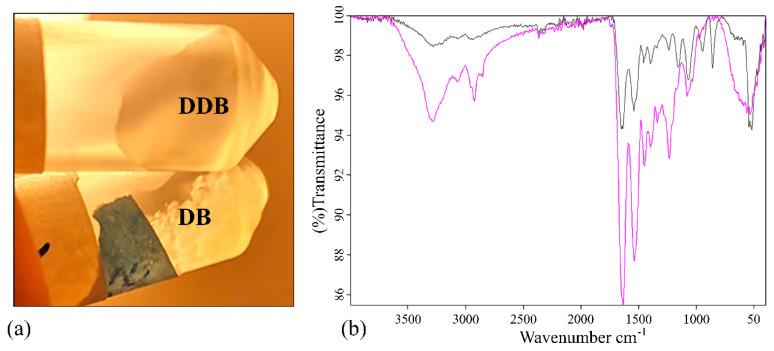
Pepsin digestion analysis. (**a**) Photograph of the samples in phosphate buffered saline stirred overnight at room temperature: (top) enzymatically digested decellularized bladder (DDB); (bottom) decellularized bladder (DB). (**b**) Fourier-transform infrared spectroscopy spectra of DB powder in black and DDB powder in pink in the region of 4000–400 cm^−1^.

The porcine urinary bladder was decellularized and enzymatically digested with pepsin. The enzymatic pepsin digestion of lyophilized DB powder (DDB), described in the Materials and Methods section, was confirmed by visual inspection (Figure 1a) and Fourier-transform infrared spectroscopy (FTIR) perform in the wavenumber range 4000–400 cm^−1^ (see Figure 1b) after dissolution in phosphate-buffered saline (PBS) solution.

The appearance of the white DB and DDB powder samples after dissolution in PBS was different (Figure 1a). The DB powder did not dissolve in PBS even after two days of vigorous shaking. However, the DDB powder completely dissolved in PBS after overnight incubation. The corresponding FTIR spectra of both samples are shown in Figure 1b. The measured FTIR spectrum of DDB sample shared similarities with the FTIR spectrum of the collagens (see Figure 1 in [37]), which can be considered as evidence of the success of the enzymatic digestion to produce a collagen-like gel from the decellularized porcine bladder powder. Both FTIR spectra exhibited characteristic IR bands which represent different vibration modes of the peptide bond. The amide A band, which is usually observed in the range 3400–3440 cm^−1^ due to the to the free N-H stretching vibration, appears at 3288 cm^−1^ in both samples due to the peak shifts to a lower wavenumber when the NH group of a peptide is part of a hydrogen bond typical of collagen-like samples [44]. The amide B bands of both samples are observed at 2923 cm^−1^, which is assignable to asymmetrical stretching of CH_2_ [45,46]. The amide I and II bands are two major bands of the protein FTIR spectrum. Thus, the amide I band, associated with the C=O stretching vibration and directly related to the polypeptide backbone conformation [45], shows strong peaks at 1646 and 1635 cm^−1^ for the DB and DDB samples, respectively. This band is a sensitive marker of peptide secondary structure [45] and it has been reported that a shift of the amine I peak to lower wavenumbers can be related to a decrease in the molecular order, suggesting the partial removal of telopeptides by pepsin during the enzymatic digestion might lead to the loss of reactive amino acids [47]. The amide III band at 1234 cm^−1^ showed much more relative intensity in the pepsin-digested gel than in the non-digested bladder powder in good agreement with the observed change of solubility of DDB with respected to DB (Figure 1a). Furthermore, both samples showed very different fingerprint regions (from 1000 to 400 cm^−1^) providing other signs of successful enzymatic digestion.

### 2.2. Rheological Properties

**Figure 2:**
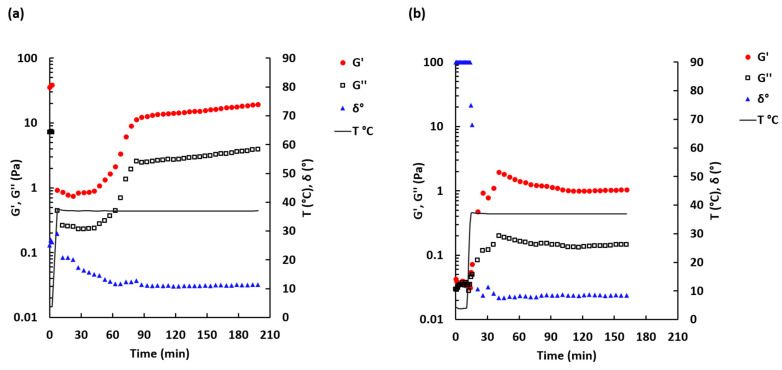
Sol-gel transition curve for 30 mg/mL DDB (**a**) and 1.5 mg/mL Matrigel (**b**) samples. Temperature was ramped from 4 to 37 °C (secondary scale). Oscillation frequency 0.5 Hz, shear strain 0.005. Mean *n* = 2–3.

Rheological characterization is an essential method employed to assess possible use of the material as injectable ECM scaffold gels. Thus, three material properties that are very important for use in the form of injectable gels are the pre-gel viscosity, which increases with protein concentration of the pre-gel [48], gelation kinetics and gel stiffness [48]. The rate of gelation is shown to be greater with increasing concentration in UBM and other ECM gels [49]. The storage modulus of the gel increases with increasing protein concentration for multiple source tissues including UBM [35,48,49] and is related to the stiffness and solid-like behavior of the gel [35]. Gelation, pre-gel viscosity and time to gelation are relevant properties for stem cell therapy. Thus, these properties can affect whether the injected gel is retained within the defect site or instead diffuses into the surrounding host tissue. Since the clinical utility of such biological gel can also be limited by its physical properties, the rheological characteristics of the enzymatically digested decellularized porcine bladder were studied and compared with Matrigel^TM^. Figure 2 shows time dependence, upon increase in temperature from 4 to 37 °C of phase angle (δ), storage modulus (G’) and loss modulus (G’’). 

The sol-gel transition time for the DDB sample (Figure 2a) was not determined because the phase angle, measured immediately after loading of the DDB sample onto the rheometer, was found to be 40.4 ± 21.4° (*n* = 4). This indicates that the sample was more likely in a transient form at 4 °C. A similar observation was made at 20 °C: the phase angle was 44.9 ± 3.0° (*n* = 2) (see Figure S1). By contrast, for the Matrigel sample, the initial phase angle was 90° and then rapidly dropped when temperature exceed 10 °C (Figure 2b), which is in agreement with the information provided by the manufacturer [50]. The phase angle for the Matrigel sample dropped within the first 7 min after temperature exceeded 10 °C. Rapid gelation at >10 °C is typical for Matrigel™ solutions [50]. For this sample, the sol-gel transition time was found to be 2.4 ± 2.1 min (*n* = 6) after temperature has reached 37 °C. 

The storage modulus G’ and loss modulus G’’ increased slowly during the first 1.5 h at 37 °C for the DDB sample and then reached a plateau level (Figure 2a). Similar but faster increases in the storage and loss moduli and reaching of plateau level were observed for the Matrigel sample after it crossed the sol-gel transition point (Figure 2b). The maximum value of G’ was 8.8 times higher for the DDB sample (19.4 ± 3.7 Pa, reached after ca. 190 min at 37 °C) compared to the Matrigel sample (2.2 ± 1.9 Pa, reached within the first 30 min at 37 °C). 

**Figure 3:**
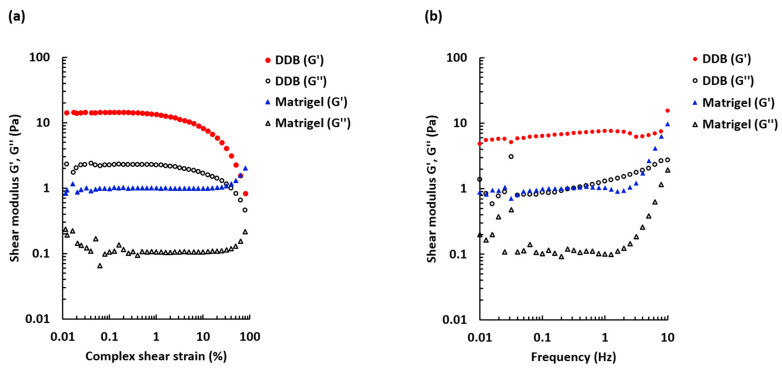
Amplitude and frequency sweep tests for 30 mg/mL DDB gel and 1.5 mg/mL Matrigel gel. (**a**) The storage modulus G’ and loss modulus G’’ dependence on shear strain over a range 0.01–100%. Oscillation frequency 1 Hz, T = 37 °C. Mean *n* = 3–4. (**b**) The frequency sweep comparing G’ and G’’ over a frequency range 0.01–10 Hz. Shear strain 0.0005, T = 37 °C. Mean *n* = 3–4.

To determine the linear viscoelastic region, an amplitude sweep test was performed (Figure 3a). 

The DDB and the reference Matrigel gels behaved differently with increased deformation. When the applied stress was beyond 1%, the DDB sample became less elastic and the G’ decreased. However, the Matrigel sample was more stable and maintained viscoelastic properties at higher strains. The results also show that the shear strains chosen for the frequency sweep measurements and gelation kinetics measurements were within the linear viscoelastic region for both DDB and Matrigel gels. Figure 3b displays frequency dependence of the storage and loss moduli for the formed gels. For the DDB sample, the storage modulus (G’) was stable over the frequency range tested (0.01–10 Hz), whereas the loss modulus was slightly increasing with increasing frequency. The storage modulus was superior to the loss modulus, confirming that the DDB sample was a true gel. Similar characteristics were found for the Matrigel reference sample, but an increase of both moduli was observed at frequencies >2 Hz

### 2.3. Cell Proliferation of ADSC 

Solubilized DDB was evaluated as a surface coating for cultivation of ADSC in vitro and compared to the Matrigel coating and without coating.

**Figure 4:**
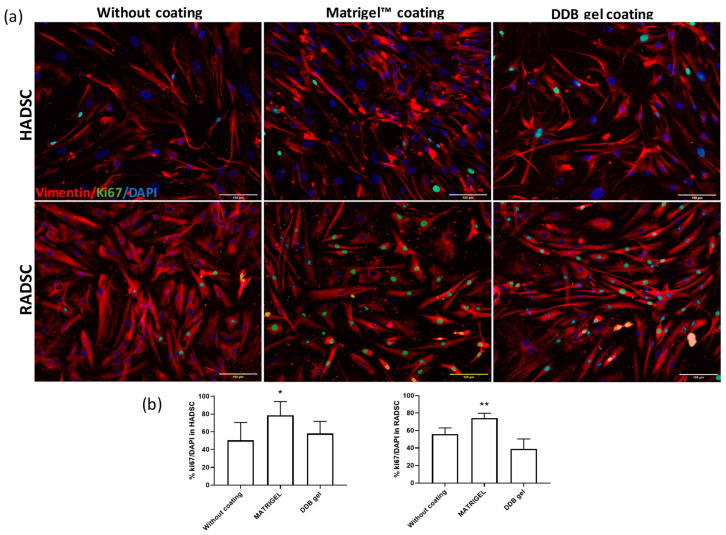
DDB coating does not accelerate cell proliferation. (**a**) Rabbit ADSC (RADSC) and human ADSC (HADSC) were grown for 72 h on coverslips without coating or covered with Matrigel or coated with DDB gel and then were subjected to double immunostaining of Ki67, a mitotic marker (green), and Vimentin, a cytoskeleton filament (red). DAPI (blue) was used for nuclei counterstaining. (**b**) Quantification of the proliferating cells measured by the percentage of Ki67 positive cells; *n* = 3; * *p* > 0.05, ** *p* > 0.01 versus without coating.

The proliferative activity and potential morphological changes were evaluated by double staining using Ki67, a mitotic marker, and vimentin, a filament that forms part of the cell cytoskeleton, of HADSC or RADSC seeded on coverslips coated with DDB gel or Matrigel or without coating (Figure 4). 

Cell proliferation was significantly higher when either RADSC or HADSC was seeded on Matrigel coated plates as expected, due to the presence of additional growth factors such as TGF- β and EGF known that promote cell proliferation [29]. The DDB gel did not show any cytotoxic effect and showed similar proliferation rates in comparison with no coating condition in both cell cultures. The HADSC and RADSC grown on the DDB gel coating did not showed any apparent difference on the expression of intracellular vimentin in comparison with any other condition; however, the shape and organization of the cells showed a more elongated shape typical of SMCs [14]. 

### 2.4. Cell Distances and Eccentricity

**Figure 5:**
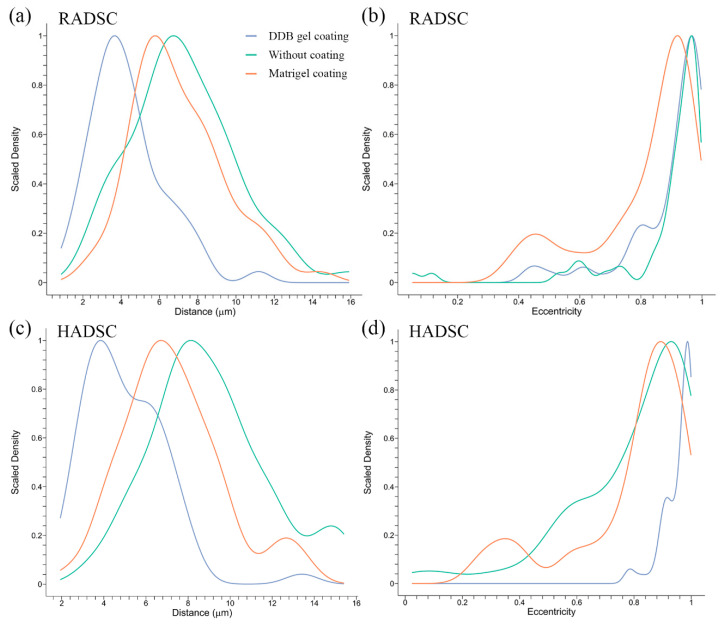
Cell-to-cell distances and eccentricity index statistical analysis of rabbit ADSC (RADSC) and human ADSC (HADSC)**.** Cells were grown for 72 h without coating (green) and with coating of Matrigel (red) or DDB bladder gel (blue): (**a**) cell distances between RADSC; (**b**) eccentricity of RADSC; (**c**) cell distances between HADSC; and (**d**) eccentricity of HADSC.

To demonstrate that the human and rabbit ADSC grown on the DDB gel coating acquire an elongated shape typical of differentiated SMCs (see Figure 4), a mathematical analysis of the microscopy images was performed. The distribution of the cell-to-cell distances were found to be significantly shorter when they are cultured on the DDB bladder gel compared to Matrigel or without coating for both RADSC (Figure 5a) and HADSC (Figure 5c) (*p* < 0.05 by the Games–Howell post-hoc test).

Cell eccentricity of RADSC (Figure 5b) and HADSC (Figure 5d) was found to be significantly increased (*p* < 0.05 by the Games–Howell post-hoc test) when the cells were gown on the DDB coating in contrast to the Matrigel coating. This indicates that both human and rabbit ADSC had an elongated shape on the bladder-derived coating. 

### 2.5. Cell Packaging Analysis 

**Figure 6:**
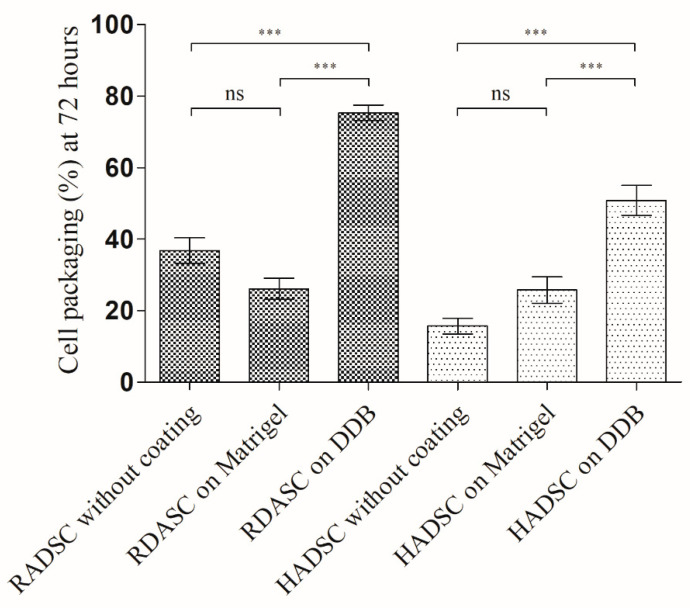
Cell packaging (%) for rabbit ADSC (RADSC) and human ADSC (HADSC): cells grown on coverslips without coating and with coating of Matrigel or DDB bladder gel for 72 h. *** *p* > 0.001; ns, not significant.

The results of the cell packaging analysis are shown in Figure 6.

Both RADSC and HADSC were more oriented and organized in bundles on the DDB gel coating compared to the other tested substrates. This increased packaging may be indicative of an onset of cellular to smooth muscle differentiation on the DDB gel coating and is in good agreement with the results shown in Figure 4 and Figure 5.

### 2.6. DDB Gel Favors Cell Smooth Muscle Maturation Stage

**Figure 7:**
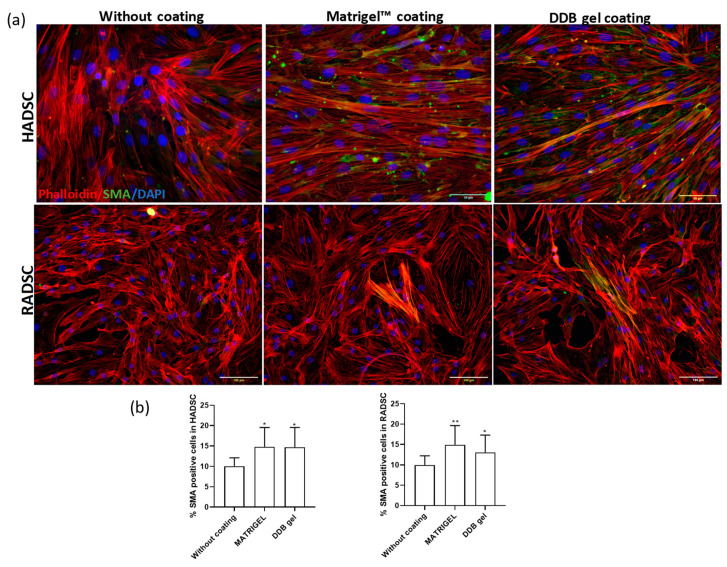
Smooth muscle actin (SMA) immune detection in rabbit ADSC (RADSC) and human ADSC (HADSC). (**a**) Cells grown on coverslips without coating or coated with Matrigel or DDB gel for one week. SMA, smooth muscle marker (green); Phalloidin (red); Dapi (blue). (**b**) Histograms showing the percent of SMA positive cells/DAPI positive cells for the tested substrates; * *p* > 0.05; ** *p* > 0.01.

The smooth muscle actin (SMA) immune detection in rabbit and human ADSC grown for one week on the coverslips without coating or with Matrigel or DDB gel coating ensured that the DDB gel and Matrigel coatings induced smooth muscle cell fate of both types of ADSC in an equivalent manner and significantly higher than in the absence of coatings (Figure 7). 

Matrigel was used as reference gel because it contains growth factors that promote proliferation and/or differentiation [29]. In fact, evidence that 3D cultures of cells on Matrigel could be beneficial for proliferation and/or differentiation has been reported [51]. For example, differentiation of myoblasts cultured in Matrigel was shown to be improved. These results are in good agreement with a previous study of a gel derived from the porcine urinary bladder matrix that supported in vitro adhesion and growth of rat aortic SMCs [35]. However, to the best of our knowledge, the myogenic differentiation of human ADSC without additional stimulation with an induction media has never been reported before.

## 3. Materials and Methods

### 3.1. Bladder Decellularization

Pig bladders were collected from a slaughterhouse. After the excess connective tissue and residual urine were removed, the bladders were consecutively washed first in bi-distilled water and then in PBS (137 mM NaCl, 2.7 mM KCl, 10 mM Na_2_HPO_4_, 1.8 mM KH_2_PO_4_; pH 7.4; Sigma Aldrich, St. Louis, MI, USA) containing 1% of streptomycin/penicillin (S/P; Invitrogen, Carlsbad, CA, USA) until blood was completely removed. For decellularization, bladders were individually transferred first into a beaker containing Tris buffer solution (10 mM, pH 8, Sigma-Aldrich) supplemented with 1% (*w/v*) sodium dodecyl sulfate (SDS; Sigma Aldrich) and incubated at 4 °C on a shaking set at 400 rpm for three days; the solution was refreshed daily. The bladders were then immersed in 0.5% (*w/v*) Triton X-100 (Invitrogen) in Tris buffer solution (10 mM, pH 8) at 4 °C for one day, and finally incubated in 0.05% (*w/v*) ammonia hydroxide (Sigma-Aldrich) solution in Tris buffer (10 mM, pH 8) in continuous shaking at 400 rpm at 4 °C for an additional day. The bladders were repeatedly washed in sterile PBS with S/P and ultrapure water containing S/P. The drained bladders were then frozen in liquid nitrogen and freeze-dried. The obtained samples of decellularized bladder are referred to as DB hereafter.

### 3.2. Enzymatic Digestion and Solubilization

The DB powder was obtained by the mechanical disruption of 300 mg of the freeze-dried DB tissue samples at 1500 rpm using a 1 cm long magnetic stir bar and a glass vial of 2 cm diameter overnight.

The obtained DB powder was enzymatically digested with 30 mg of pepsin (10108057001 Sigma-Aldrich) dissolved in 30 mL of 0.1 N HCl at optimal operating pH (1.8–2.2) in continuous shaking at 400 rpm at room temperature for 48 h. The enzymatic reaction was blocked by neutralizing the pH to 7.4 with 1 N NaOH. The solution was then balanced with 10× PBS (1:10). Non-digested fragments were removed by filtering with a cell strainer of 70 mm (352350 Falcon) and the remaining solution was sterilized by UV irradiation for 10 min. The concentration of the final gel was adjusted to its optimal concentration (30 mg/mL). These obtained sample of enzymatically digested decellularized bladder will be named DDB hereafter. The DDB gel was generated by incubation at 37 °C for 90 min. All solutions should be used in cold to ensure the highest efficiency of the gelification.

### 3.3. Fourier Transform Infrared Spectroscopy

FTIR spectra of dried DB and DDB samples were analyzed with a Bruker Alpha FTIR spectrophotometer (Billerica, MA, USA). FTIR spectra were acquired in transmission mode in the wavenumber range of 4000 to 400 cm^−1^ after 32 scans at a resolution of 2 cm^−1^.

### 3.4. Rheological Characterization

DDB powder solution was prepared by dissolving 4.5 mg of the sample in 150 µL PBS (3 mM, pH 7.4; Gibco^TM^ Thermo Fisher Scientific, Waltham, MA, USA) under stirring at room temperature until the sample is fully dissolved, and then cooled down on ice to ca. 4 °C. Phenol Red Free Matrigel Basement Membrane Matrix (REF 356237, Corning Inc., New York, NY, USA) was dissolved in ice-cold PBS to obtain the 1.5 mg/mL standard concentration and was stored on ice prior to measurements. Cold solutions were loaded onto the prechilled lower plate (4 °C) of the Kinexus Pro+ (Malvern, UK) rheometer equipped with 2 cm plate geometry probes. The gap between the probes was fixed to 0.1 mm. A solvent trap was used to prevent sample drying. To measure gelation kinetics, the development of phase angle, storage modulus (G’) and loss modulus (G’’) was followed as a function of time at 0.5 Hz frequency oscillation and strain 0.005. The first 3–10 measurements were done at 4 °C, and then temperature was ramped from 4 to 37 °C at rate of 10 °C/min and held at 37 °C during approximately 3 h until G’ reached an equilibrium. To determine the viscoelastic region of the gels, the strain sweep, at a constant frequency of 1 Hz, was performed. To characterize the viscoelastic properties as a function of frequency, a frequency sweep at constant strain 0.0005 was carried out. The data were acquired and analyzed using the rSpace^®^ software (Malvern Instruments).

### 3.5. Human and Rabbit ADSC Isolation

Human adipose tissue was obtained from surplus fat tissue during knee prosthesis surgery of four patients under sterile conditions. As exclusion criteria, no samples were collected from patients with a history of cancer or infectious diseases at the time of the surgery (viral or bacterial). All human patients voluntarily signed an informed consent document for the use of the adipose samples. The human samples were anonymized, and the experimental procedure was previously evaluated and accepted by the Regional Ethics Committee for Clinical Research with Medicines and Health Products following the Code of Practice 2014/01. Rabbit adipose tissue was collected as a surplus tissue from one single individual, a female New Zealand rabbit (8 months old), shortly before sacrifice in the previous study [52]. Both HADSC (human ADSC) and RADSC (Rabbit ADSC) were isolated, expanded and characterized as previously described [53,54]. Briefly, cells were maintained and expanded in DMEM supplemented with 10% (*w/v*) fetal bovine serum, 2 mM l-glutamine, 1% l-glucose and P/S. ADSC at passages 3–4 were used for the biological assays.

### 3.6. Cell Proliferation and Differentiation Analysis

Glass coverslip (18 cm^2^) without coating or coated with 1.5 mg/mL of Matrigel or coated with the 30 mg/mL DDB gel were placed into 12-well plates. The cells of HADSC or RADSC were distributed on top of the coverslips (1000 cells/coverslip) in growth culture medium for 24 h, 72 h or one week of incubation. Three independent experiments were performed in triplicates. Then, cells were fixed in 4% (*w/v*) paraformaldehyde for 10 min, washed and permeabilized with 0.1% (*w/v*) Triton X-100. For immunostainings, the samples were blocked with 5% (% *v/v*) normal goat serum in PBS. The primary antibodies were incubated overnight at 4 °C: vimentin (1:500 ab8978, Abcam, Cambridge, MA, USA), Ki67 (1:500 ab15580, Abcam, Cambridge, MA, USA) and anti-SMA (A5228, Sigma-Aldrich, St. Louis, MO, USA). After washing, the samples were incubated with the secondary antibodies (Alexa-488 or Alexa-555 for mouse or rabbit primary antibodies) at room temperature for 2 h. Phalloidin conjugated with Alexa Fluor 647 (P1951, Sigma-Aldrich) was used at 1:1000 dilution and incubated with the samples for 1 h for F-actin cytoskeletum staining. All cells were counterstained by incubation with 4,6-diamidino-2-phenylindole dihydrochloride (DAPI; Invitrogen). The percentage of ki67 or SMA positive cells was evaluated after image acquisition in a Leica fluorescent microscope in triplicates. The cell size analysis of at least 60 cells from vimentin positive cells analysis was performed using ImageJ/Fiji software. Consistent exposures were applied for all images.

### 3.7. Mathematical Analysis of Microscopy Images

The ImageJ software was used to measure several cell characteristics for the three types of cell culture substrates: without coating, coating of Matrigel and coating of DDB. Specifically, the distance between cells (in μm), cell width (in μm) and cell length (in μm) were measured.

The lengths of the semi-major and semi-minor axes corresponding to each cell were used to approximate the cell’s eccentricity. The eccentricity of a cell, *e*, was computed with Equation (1):

(1) $$e=\sqrt{1-\frac{b^{2}}{a^{2}}}$$

where *a* and *b* are the lengths of the semi-major and semi-minor axes, respectively, of the ellipse that better fits the cell’s shape. The eccentricity ranges from 0 to 1, where values closer to 0 indicate a high degree of roundness (*e* = 0 corresponds to a circle) and values closer to 1 indicate the opposite (a highly elongated shape).

As SMCs tend to orient and organize into bundles, an increased packaging may be indicative of an onset of cellular to smooth muscle differentiation. Therefore, cell packaging analysis for RADSC and HADSC grown without coating and with coating of Matrigel or DDB was performed from at least 150 cells observed in the microscopy images.

### 3.8. Statistical Analysis

The Brown–Forsythe test [55] was used to assess the differences in cell characteristics grown on the different culture substrates. This test was chosen because it does not require the assumption of equal variances, which was consistently violated by the available data. In addition, the Games–Howell post-hoc test [56] was used for evaluating two-by-two comparisons between culture media. The results of cell proliferation and SMA immune detection were statistically compared by ANOVA followed by multiple Tukey’s post-hoc analysis by the GraphPad Prism 6 software at significance level of at least *p* < 0.05.

## 4. Conclusions

An injectable porcine bladder-derived gel with suitable rheological properties was prepared in this study. The gel was produced with pepsin-digested decellularized bladder powder (DDB) dissolved in PBS. The DDB solution was in a transient form at room temperature and the gel formation was completed after 1.5-h incubation. Our results show that the proliferation and morphology of the cells grown on the DBB gel coating were different from those grown on Matrigel coating. This DDB gel was shown to be able to induce in vitro cell differentiation of rabbit- and human-derived stem cells into smooth muscle cells without using any induction media or external stimulation. After this study, whether this stem cell differentiation induction also occurs in other similar gels produced from bigger or smaller animal bladders remains an open question which requires further investigation. This DDB gel in combination with ADSC is a very promising platform that could be able to reconstruct the detrusor muscle part of the human urinary bladder, among many other potential applications.

## Supplementary Materials

Supplementary Materials can be found at https://www.mdpi.com/1422-0067/21/22/8608/s1. Figure S1: Sol-gel setting curve obtained for the solution of the decellularized bladder (DDB) at 4 and 20 °C.
