# Supplementary material for: Injectable Gel Form of a Decellularized Bladder Induces Adipose-Derived Stem Cell Differentiation into Smooth Muscle Cells In Vitro
Source: Int J Mol Sci. 2020 Nov 15;21(22):8608. doi: 10.3390/ijms21228608 (PMC7696281; doi:10.3390/ijms21228608)
Supplement: Supplementary file 1 [file ijms-21-08608-s001.pdf]

Supplementary Materials

Victoria Moreno-Manzano [1,*], Daria Zaytseva-Zotova [2], Eric López-Mocholí [1], Álvaro Briz-Redón [3], Berit Løkensgard Strand [2] and Ángel Serrano-Aroca [4,*]

[1] Neuronal and Tissue Regeneration Lab, Centro de Investigación Príncipe Felipe, c/Eduardo Primo Yúfera, 3, Valencia 46012, Spain
[2] NOBIPOL, Department of Biotechnology and Food Science, NTNU Norwegian University of Science and Technology, Sem Sælands vei 6-8, N-7491 Trondheim, Norway.
[3] Statistics Office, City Council of Valencia, plaza Ayuntamiento 1, 46002 Valencia, Spain.
[4] Biomaterials and Bioengineering Lab, Centro de Investigación Traslacional San Alberto Magno, Universidad Católica de Valencia San Vicente Mártir, c/Guillem de Castro 94, 46001 Valencia, Spain.
* Correspondence: vmorenom@cipf.es, Tel: +34 963289681 Ext. 1103 (V.M-M) ; angel.serrano@ucv.es, Tel: +34 963637412 Ext. 5256 (Á.S-A)

Received: date; Accepted: date; Published: date

The sol-gel setting curve obtained for the solution of the decellularized bladder (DDB) at 4 and 20°C is shown in Figure S1.

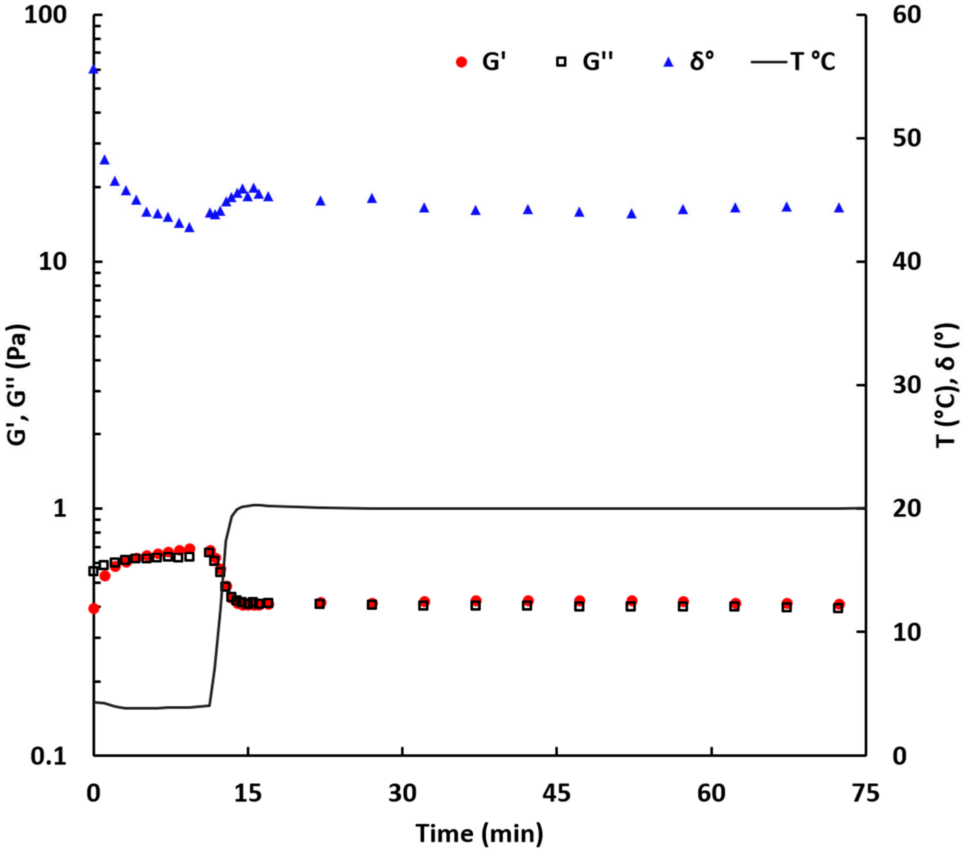

Figure S1. Sol-gel setting curve obtained for the solution of the decellularized bladder (DDB) at 4 and 20°C

Author Contributions: Conceptualization, V.M-M, D.Z-Z., BLS and Á.S-A ; methodology, V.M-M, D.Z-Z., BLS and Á.S-A ; software, V.M-M, D.Z-Z., A.B-R. and Á. S-A. ; validation, V.M-M, D.Z-Z., BLS, D.Z-Z. and Á.S-A; formal analysis, V.M-M, D.Z-Z., BLS and Á.S-A; investigation, V.M-M, D.Z-Z., E. L-M., A.B-R., BLS and Á.S-A; resources, V.M-M, BLS and Á.S-A; data curation, V.M-M, D.Z-Z., A.B-R., BLS and Á.S-A; writing—original draft preparation, Á.S-A; writing—review and editing, V.M-M, D.Z-Z., A.B-R., BLS and Á.S-A; visualization, V.M-M, D.Z-Z., A.B-R. and Á.S-A; supervision, V.M-M, BLS and Á.S-A; project administration, Á.S-A; funding acquisition, V.M-M and Á.S-A.

Funding: This research was funded by the Regional Ministry of Health of the Valencian Community (Generalitat Valenciana), grant AEI "RTI2018-095872-B-C21/ERDF" and by the Fundación Universidad Católica de Valencia San Vicente Mártir, grants 2019-231-003UCV and 2020-231-001UCV.

Conflicts of Interest:

The authors declare no conflict of interest.
